# Assessing Palliative Care Needs in Nursing Homes Among Residents With Cognitive Impairment: UPLIFT Trial Findings

**DOI:** 10.1093/geroni/igaf122.3370

**Published:** 2025-12-31

**Authors:** John Cagle, Peiyuan Zhang, Todd Becker, Alex Floyd, Tracie Schwoyer-Morgan, Becky Ridder, Kathleen Unroe

**Affiliations:** University of Maryland, Batimore, Maryland, United States; University of Maryland, Baltimore County, Baltimore, Maryland, United States; WashU Medicine, St. Louis, Missouri, United States; Regenstrief Institute, Indianapolis, Indiana, United States; Gilchrist, Baltimore, Maryland, United States; Eskenazi Health, Indianapolis, Indiana, United States; Indiana University School of Medicine, Indianapolis, Indiana, United States

## Abstract

Palliative care (PC) has been shown to improve quality of care and life for nursing home (NH) residents. The PC needs and acuity of NH residents, particularly those with dementia, remain understudied. Using data from a large, NIA-funded trial evaluating PC in NHs, 655 residents with cognitive impairment were enrolled from 16 NHs across two states. NH staff, typically social workers and nurses, were trained as in-house PC leads. PC leads were asked to complete a PC screening on all enrolled residents to: identify needs; determine whether a PC consult is needed; and, if so, determine triage status for a consult visit. PC screening descriptives are presented and predictors of triage status were modeled. Initial screens were completed for 528 residents (80.6%) of all enrolled residents. Reasons for not being screened were resident discharge (e.g., to hospital) and death. Among six possible PC needs, 15% of residents had no identified needs, 42% had one need, and the remainder (42.8%) had ≥2 needs. The most prevalent needs were polypharmacy (58.1%), a lack of goals-of-care documentation (26.5%), functional decline/medical complexity (24.8%), unmanaged symptoms (19.7%), incongruent care (6.3%), and recent hospitalization (4.5%). Among those screened, a majority (n = 461, 87.3%) indicated a PC consult, with 5.4% of those cases being triaged as high priority. A greater number of needs (B=.42), polypharmacy (B=.33), and unmanaged symptoms (B=.31) significantly predicted higher triage priority (p<.001). Understanding residents’ PC needs can inform tailored policies and practices to address such gaps in care.

